# The mouse Gene Expression Database (GXD): 2021 update

**DOI:** 10.1093/nar/gkaa914

**Published:** 2020-10-26

**Authors:** Richard M Baldarelli, Constance M Smith, Jacqueline H Finger, Terry F Hayamizu, Ingeborg J McCright, Jingxia Xu, David R Shaw, Jonathan S Beal, Olin Blodgett, Jeffrey Campbell, Lori E Corbani, Pete J Frost, Sharon C Giannatto, Dave B Miers, James A Kadin, Joel E Richardson, Martin Ringwald

**Affiliations:** The Jackson Laboratory, 600 Main Street, Bar Harbor, ME 04609, USA; The Jackson Laboratory, 600 Main Street, Bar Harbor, ME 04609, USA; The Jackson Laboratory, 600 Main Street, Bar Harbor, ME 04609, USA; The Jackson Laboratory, 600 Main Street, Bar Harbor, ME 04609, USA; The Jackson Laboratory, 600 Main Street, Bar Harbor, ME 04609, USA; The Jackson Laboratory, 600 Main Street, Bar Harbor, ME 04609, USA; The Jackson Laboratory, 600 Main Street, Bar Harbor, ME 04609, USA; The Jackson Laboratory, 600 Main Street, Bar Harbor, ME 04609, USA; The Jackson Laboratory, 600 Main Street, Bar Harbor, ME 04609, USA; The Jackson Laboratory, 600 Main Street, Bar Harbor, ME 04609, USA; The Jackson Laboratory, 600 Main Street, Bar Harbor, ME 04609, USA; The Jackson Laboratory, 600 Main Street, Bar Harbor, ME 04609, USA; The Jackson Laboratory, 600 Main Street, Bar Harbor, ME 04609, USA; The Jackson Laboratory, 600 Main Street, Bar Harbor, ME 04609, USA; The Jackson Laboratory, 600 Main Street, Bar Harbor, ME 04609, USA; The Jackson Laboratory, 600 Main Street, Bar Harbor, ME 04609, USA; The Jackson Laboratory, 600 Main Street, Bar Harbor, ME 04609, USA

## Abstract

The Gene Expression Database (GXD; www.informatics.jax.org/expression.shtml) is an extensive and well-curated community resource of mouse developmental gene expression information. For many years, GXD has collected and integrated data from RNA *in situ* hybridization, immunohistochemistry, RT-PCR, northern blot, and western blot experiments through curation of the scientific literature and by collaborations with large-scale expression projects. Since our last report in 2019, we have continued to acquire these classical types of expression data; developed a searchable index of RNA-Seq and microarray experiments that allows users to quickly and reliably find specific mouse expression studies in ArrayExpress (https://www.ebi.ac.uk/arrayexpress/) and GEO (https://www.ncbi.nlm.nih.gov/geo/); and expanded GXD to include RNA-Seq data. Uniformly processed RNA-Seq data are imported from the EBI Expression Atlas and then integrated with the other types of expression data in GXD, and with the genetic, functional, phenotypic and disease-related information in Mouse Genome Informatics (MGI). This integration has made the RNA-Seq data accessible via GXD’s enhanced searching and filtering capabilities. Further, we have embedded the Morpheus heat map utility into the GXD user interface to provide additional tools for display and analysis of RNA-Seq data, including heat map visualization, sorting, filtering, hierarchical clustering, nearest neighbors analysis and visual enrichment.

## INTRODUCTION

The longstanding objective of GXD has been to capture and integrate different types of mouse developmental expression information, with a focus on endogenous gene expression in wild-type and mutant mice, and to make these data readily accessible to researchers via biologically- and biomedically-relevant searches. As an integral component of the larger Mouse Genome Informatics (MGI) resource (1–3), GXD combines expression data with genetic, functional, phenotypic and disease-oriented data, thereby enabling unique and powerful search capabilities that foster insights into the molecular mechanisms of human development, differentiation, and disease. In addition, GXD maintains links to external expression resources, including gene-based links to expression data from other vertebrate model organisms that are highly relevant for developmental research: zebrafish (ZFIN; 4), *Xenopus* (Xenbase; 5) and chicken (GEISHA; 6). For many years, GXD has collected RNA and protein expression information from RNA *in situ* hybridization, immunohistochemistry, *in situ* reporter (knock in), RT-PCR, northern blot, and western blot experiments. These complex, heterogeneous expression data, generated by many laboratories and distributed through thousands of publications, are acquired through curation of the scientific literature and by collaborations with large-scale expression projects. GXD curators annotate these data in standardized ways by making extensive use of controlled vocabularies and ontologies (7–10). Since our last report in the NAR Database issue (2), we have (i) continued to curate these classical types of expression data, (ii) expanded GXD to represent RNA-Seq and microarray experiments in searchable form, (iii) begun to fully integrate RNA-Seq data into GXD and into the larger MGI system, and (iv) developed new user interface utilities for the display and analysis of expression data. In the following, we will report on our progress, putting the main emphasis on the description of new types of data and new search and display capabilities in GXD.

## PROGRESS IN DATA ACQUISITION FOR CLASSICAL TYPES OF EXPRESSION DATA

### Comprehensive literature survey

We systematically survey journals to find all publications examining endogenous gene expression during mouse development. In a first curation step for each paper, we annotate the genes and ages analyzed and the expression assay types used. Annotations are based on the entire publication, including Supplemental Data, and employ official nomenclature for genes. This information, combined with bibliographic information from PubMed, is accessible via the Gene Expression Literature Search form (http://www.informatics.jax.org/gxdlit). GXD’s literature content records are comprehensive and up-to-date from 1990 to the present. GXD has records for >28 400 references and >16 200 genes. The Gene Expression Literature Search provides scientists with an effective tool for finding publications with specific expression data, and it helps GXD Curators to prioritize papers for detailed expression annotation.

### Detailed expression data

In a second curation step, the expression data are annotated in detail. For each expression assay, we record information about the gene studied, the strength and pattern of expression in specific anatomical structures, the probes and experimental conditions used, and the age and genetic background of the specimen(s) analyzed. Images of the data accompany the annotations when available. Standard gene, mouse strain and allele nomenclature, controlled vocabularies and an extensive anatomy ontology are employed to enable thorough data integration and search capabilities. See http://www.informatics.jax.org/assay/MGI:2673718 for an example of an RNA *in situ* record, and Figure 4C for an example of immunohistochemistry records (partial records shown). As of September 2020, GXD contains detailed expression data for nearly 15 000 genes, including data from numerous strains of wild-type mice and from >4900 mouse mutants. GXD now holds >365 000 images and >1.74 million expression result annotations for classical types of expression data. The majority of these data (85%) are from *in situ* hybridization, immunohistochemistry and *in situ* reporter (knock in) experiments.

## PROGRESS IN DATA ACQUISITION FOR HIGH-THROUGHPUT EXPRESSION DATA

### RNA-Seq and expression microarray metadata index

GXD now provides a searchable metadata index of mouse high-throughput expression experiments available in public repositories (11). We incorporate mouse RNA-Seq and expression microarray experimental metadata from the Gene Expression Omnibus (GEO) (12) and ArrayExpress (13), and apply GXD annotation standards to samples and attributes of experiments that meet GXD’s scope of endogenous expression. This includes experiments that examine endogenous expression in wild type and mutant mice, and cover the entire life span of the laboratory mouse (all pre- and postnatal stages). Researchers can now find comprehensive sets of high-throughput experiments using standardized search terms from controlled vocabularies and ontologies. This task is not possible when conducting searches through repository resources, since standardized metadata are not required for data submission, and term heterogeneity is widespread.

**Figure 1.**
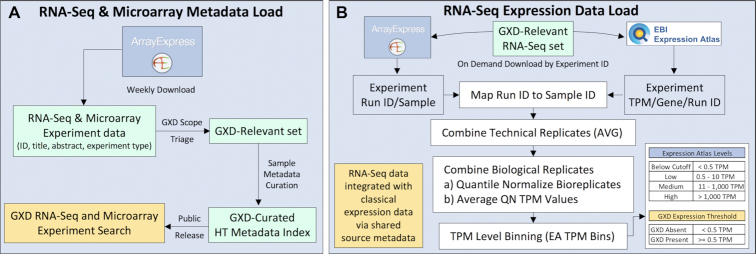
Overview of GXD high-throughput expression data loads. (**A**) GXD loads RNA-Seq and microarray metadata from ArrayExpress weekly, to download experiment-level information for new mouse RNA-Seq and expression microarray experiments. Experiments are evaluated for GXD relevance. Sample metadata from GXD-relevant experiments are downloaded on demand into a GXD high-throughput sample curation tool, where sample metadata and experimental variables are annotated using controlled vocabularies and ontologies, and added to the GXD high-throughput expression metadata index, released weekly. (**B**) The GXD RNA-Seq Expression Data load runs on demand as new GXD-relevant RNA-Seq experiments become available from the Expression Atlas (EA). Per experiment, technical replicate identifiers (Run IDs) per Sample are loaded from ArrayExpress and TPM values per gene, per Run ID are loaded from the Expression Atlas. The load joins technical replicate information to produce TPM values per gene, per run ID, per sample for each experiment. TPM values per gene from technical replicates (run IDs for a sample) are averaged. Biological replicates are identified by shared GXD-curated metadata source profiles, and combined in a two step process of (A) quantile normalizing TPM values across bioreplicates for an experiment and (B) averaging quantile normalized (QN) TPM values for each gene of the bioreplicate set. These averaged, QN TPM values are then mapped to TPM range bins established by the Expression Atlas, and to a GXD expression threshold (Present/Absent), based on TPM value relative to the Below Cutoff value range. RNA-Seq expression data are integrated with classical types of GXD expression data through the combination of shared biological source metadata, shared gene annotations and common use of Present/Absent calls.

We load mouse experiment-level metadata (ID, title, abstract and experiment type) from ArrayExpress weekly (Figure 1A). A dedicated curation tool facilitates efficient experiment evaluation and sample and experiment attribute annotation to standardized terms (not shown). An initial triage step identifies experiments that are considered consistent with GXD scope. Manual evaluation at this step is supported by supervised machine learning (a linear SVM classifier, see (11) for more detail) which has proven to be effective for the classification of out-of-scope experiments. Sample metadata for GXD-relevant experiments are downloaded on demand into the curation tool, annotated to standard terms and added to the index. Of 16 782 high-throughput expression experiments downloaded from ArrayExpress, 3163 were considered relevant to GXD’s scope (2463 microarray, 700 RNA-Seq). At the time this load was developed, ArrayExpress included all experiments from GEO. This is no longer the case. GXD plans to extend this load to acquire data directly from both ArrayExpress and GEO, assuring complete representation from both repositories.

### RNA-Seq expression data in GXD

GXD has incorporated high quality RNA-Seq expression data for GXD-relevant RNA-Seq experiments in GXD’s RNA-Seq and Expression Microarray Metadata Index. These data are imported from the Expression Atlas at EBI (14), and then processed further by GXD to allow full integration into our system, and accessibility through our search and display tools. The Expression Atlas incorporates primary data for high-quality RNA-Seq data sets from ArrayExpress and GEO, and applies a processing pipeline designed to leverage state-of-the-art annotation methods to generate reliable, updated TPM values for genes represented in current Ensembl releases (14). We download these TPM values, and prepare the data for integrated access using GXD’s RNA-Seq processing pipeline (Figure 1B). Expression data for new GXD-relevant RNA-Seq experiments from the Expression Atlas are incorporated into GXD as they become available.

**Figure 2.**
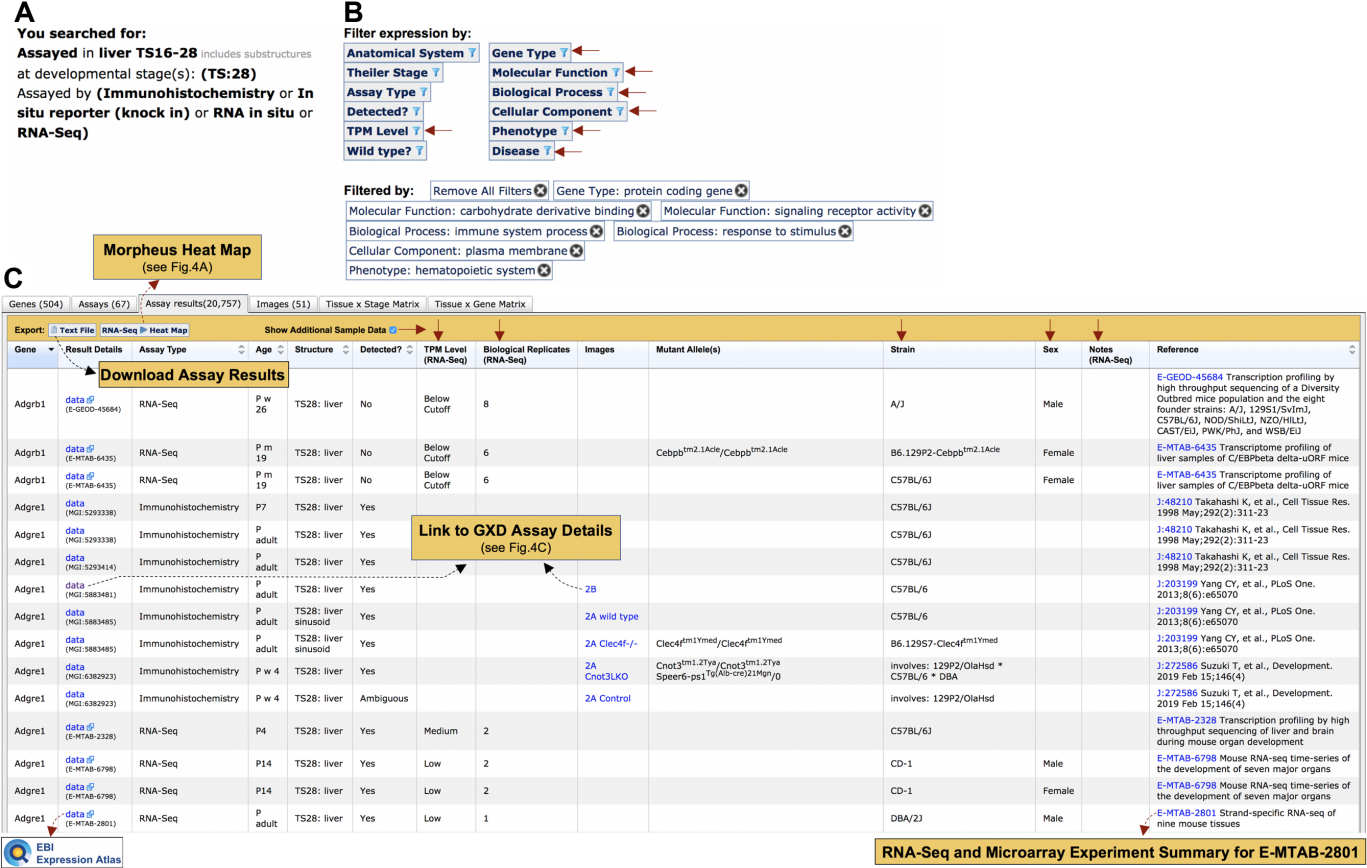
Integrated Classical and RNA-Seq Expression Results. Partial results from a search for genes expressed in liver, at TS:28 (postnatal), assayed by classical *in situ* methods and by RNA-Seq are shown. New features are identified with red arrows, featured links shown as dotted arrows. (**A**) Search parameters. (**B**) Suite of expression result filters. Sample-level filters (left column) restrict expression results by sample metadata, and include the new TPM Level filter (RNA-Seq data only). New gene-level filters (right column) restrict expression results by gene annotations, including gene type, and high-level terms for ontology annotations (Gene Ontology, Mammalian Phenotype Ontology, and Disease Ontology). Filters enacted for this result set are shown (Filtered by). (**C**) Filtered result set showing Assay results tab (Assays tab view shown in Figure 3). Link-outs indicated by dotted arrows. New sample-level metadata columns (down arrows) can be shown/hidden with the Show Additional Sample Data toggle (RNA-Seq data-specific columns indicated). A new Morpheus heat map of RNA-Seq results is rendered by clicking the RNA-Seq ▸ Heat Map button (see Figure 4A). New links to the Expression Atlas at EBI and the GXD RNA-Seq and Microarray Experiment Summary for RNA-Seq experiments are shown. Links to the GXD assay details (including images) exhibited in Figure 4C, from the Result Details and Images columns are shown. The complete Assay results set can be exported as text to the user's desktop, and includes the new sample-level metadata columns shown.

A primary goal of GXD’s RNA-Seq processing pipeline is to identify the unique biological replicate sets for each experiment; determine the averaged quantile normalized TPM value for each gene per biological replicate set; and assign each of these TPM values a Present/Absent call in GXD. RNA-Seq expression data files from the Expression Atlas provide TPM values per gene for each technical replicate (run) of a given experiment (TPM/gene/runID/experiment). The samples from which technical replicates are derived (often multiple runs per sample) are not specified in the TPM data files. The GXD metadata index, however, is annotated at the sample level (technical replicate identifiers are not included in the index). Thus, to join GXD-curated sample metadata with Expression Atlas TPM values, a second load of run IDs per sample for each experiment is necessary from ArrayExpress (see Figure 1B). TPM values per gene from technical replicates are averaged to account for any technical variation. Then, to lessen the influence of biological sampling variation, and to avoid excessive redundancy in GXD user interface displays, sample-level TPM values for biological replicates are combined. Since biological replicate information is not provided in the files downloaded from the Expression Atlas or ArrayExpress, we take advantage of GXD-curated metadata to identify bioreplicate samples for each experiment. Samples from the same experiment that share all metadata field values are considered bioreplicates. Bioreplicate sample TPM values for each gene are consolidated in a two-step process. For each experiment, sample-level TPM values for bioreplicate samples are first quantile normalized (15) using python numpy in the Pandas DataFrame, and then these quantile normalized (QN) TPM values are averaged for each gene across the bioreplicate sample set. This results in a single TPM value per gene per set of sample bioreplicates. We store bioreplicate set information in the database and assign each bioreplicate set a unique identifier (see Figure 4A). Finally, we map averaged QN TPM values to the same TPM range bins used by the Expression Atlas (see Figure 1B), and assign a Present/Absent value based on TPM values relative to the Below Cutoff value range (<0.5 TPM = Absent expression in GXD). GXD uses the same Present/Absent values for classical expression assays, thus this last step allows us to fully integrate RNA-Seq data with classical expression data in GXD searches, filters and displays (see Figure 2). To date, we have loaded expression data for 70 RNA-Seq experiments in the GXD metadata index. These include 1846 distinct samples that were condensed to 631 biological replicate sets, representing 88 distinct anatomical structures and 85 distinct mouse strains. Mouse genes represented in Expression Atlas RNA-Seq TPM files cover the entire Ensemble transcriptome (nearly 55 000 genome features), including protein-coding and non-coding RNA genes. Total RNA-Seq assay results amount to nearly 35 million, with comprehensive genome coverage for each experiment.

## IMPROVEMENTS TO THE GXD USER INTERFACE

### RNA-Seq and microarray experiment search

The RNA-Seq and Microarray Experiment Search tool [http://www.informatics.jax.org/gxd/htexp_index; detailed in (11)] enables users to quickly and reliably find specific high-throughput expression data sets from the metadata index described above. The search form supports detailed queries for RNA-Seq and/or transcription microarray experiments by attributes of the samples assayed, including anatomical structure, developmental stage, mutant gene, mouse strain and sex. The search form also provides a text search function that returns experiments by text string matches in titles or descriptions. In addition, users can search for specific experiments by experiment ID.

The search results summary provides well organized information profiles of each experiment returned, which include title and full description, assay type (method), study type and experimental variables involved (i.e. the metadata fields that distinguish samples in the study). Access to GXD-curated metadata details for each sample of an experiment is prominent on the experiment summary. The total number of samples is shown and the number of samples that matched the search parameters is also shown. This is a valuable feature, since experiments can include samples from diverse biological sources. A link is provided to a tabular view of all samples for an experiment with their associated metadata, and the samples that matched search parameters are distinguished. When experiments are returned by text string match, matching text is conveniently highlighted. The summary also includes links to relevant external resources for each experiment where available, including PubMed, ArrayExpress, GEO and the Expression Atlas. Finally, for RNA-Seq experiments included in GXD’s RNA-Seq expression data load, links are provided to the GXD expression summary page for the corresponding experiment.

### New and integrated search capabilities for RNA-Seq and classical expression data

Our approach is to add value to RNA-Seq data by integration with the other expression data types in GXD, and with the genetic, functional, phenotypic and disease-oriented data in MGI. This integration allows us to provide new search and analysis capabilities for RNA-Seq data. The newly incorporated RNA-Seq data are accessible throughout much of the GXD user interface, including (a) the Standard Gene Expression Data Search, which permits querying for expression data by an array of different search parameters, including user-specified metadata and biomedically relevant annotation profiles (http://www.informatics.jax.org/gxd; Standard Search tab); (b) the GXD Batch Search tool, which returns expression data from input lists of genes (http://www.informatics.jax.org/gxd/batchSearch) and (c) the Mouse Developmental Anatomy Browser, where links to expression data from specific anatomical structure searches are superimposed on a dynamic hierarchical display of mouse anatomical structures (http://www.informatics.jax.org/vocab/gxd/anatomy/). RNA-Seq data are excluded from the default settings of the Standard Gene Expression Data Search due to the expansive result sets common to genome-wide assays; this is clearly indicated on the Search Form. RNA-Seq data can be included with a single click in the Assay types section on the form. To assure responsive system performance, we optimized front end configuration and imposed reasonable limits for Standard and Batch GXD searches. For the Standard Gene Expression Data search, we set a limit of 21 million expression assay results. This threshold is high enough to include all results for any anatomical structure or developmental stage spanning all assay types (including RNA-Seq). The interface informs users when their search results pass this threshold, and encourages search refinement. The GXD Batch Search has an input limit of 5000 genes.

All of GXD’s expression data search forms, and links provided from the Gene Expression section of MGI Gene Detail pages lead to the same multi-tabbed displays that summarize data at different levels of detail (Genes, Assays, Assay results, Images) and via two different Matrix Views (Tissue × Stage and Tissue × Gene). RNA-Seq data are integrated into these summary views when selected on the Standard search form. Tools provided on these summaries allow users to filter, sort, and iteratively refine search results, and download the data for further analysis. We have recently added new gene-level filters that enable users to narrow search results effectively (see Figure 2B).

Figure 2 shows results of a search that would be of interest to researchers who study macrophage function in the liver. Search parameter details are displayed in the upper left of the summary (Figure 2A), and users have the option to open the query form and modify their search at any time. A suite of filters that allows refinement of expression results is shown in Figure 2B. A new quantitative expression filter that restricts RNA-Seq data sets by TPM Level was added to pre-existing expression result filters (left-hand column). Six new gene-level filters (right-hand column) have also been added that greatly expand analytical capacity, as users can restrict expression results to sets of genes by gene type and by annotations to high-level terms of the Gene Ontology (GO, 16), Mammalian Phenotype Ontology (MP, 17) or Disease Ontology (DO, 18) (Figure 2B). Separate filters are combined by Boolean AND, while multiple choices within a filter are combined by Boolean OR. Expression results from liver were refined by applying a set of gene-level filters designed to target genes that have GO and MP annotations consistent with macrophage function (see Filtered by list in Figure 2B). These filters reduced the initial assay result set by over 100-fold, by dropping the genes returned from 54 972 (not shown) to 504.

**Figure 3.**
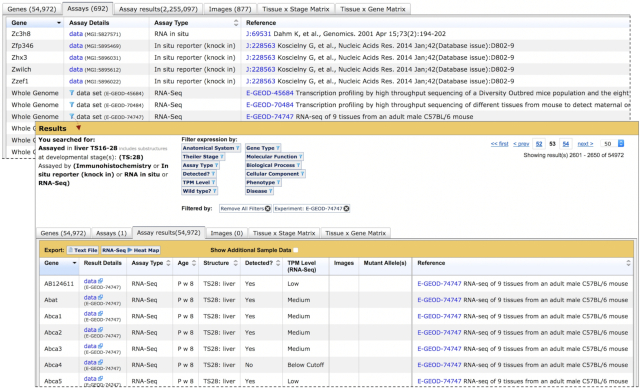
Access to Integrated Results for Specific RNA-Seq Experiments from the Assays Tab. Assays tab view (upper table) of expression results for RNA-Seq and *in situ* assays of mouse liver at TS:28. For classical experiment types, each gene analyzed and the assay type used are specified for each assay, and links are provided to GXD assay details (data) in the Assay Details column. For RNA-Seq experiments, ‘Whole Genome’ is displayed in the Gene column, because each experiment involves all genes in the Ensembl transcriptome. The default sort for classical expression type assays is by gene symbol. Whole Genome RNA-Seq experiments are positioned after classical type assays, and are sorted by experiment ID. The transition between classical type and RNA-Seq assays is shown in the upper table. Users can view integrated expression results for an individual RNA-Seq experiment by clicking on the filter icon in the Result Details column for that experiment. This applies a filter to the original search, which limits results to the selected RNA-Seq experiment (lower table). The full suite of annotation filters is now available to explore results from that single experiment. Links to complete expression results (without search restrictions) for a single RNA-Seq experiment can be obtained from the GXD RNA-Seq and Microarray Experiment Summary (not shown).

**Figure 4.**
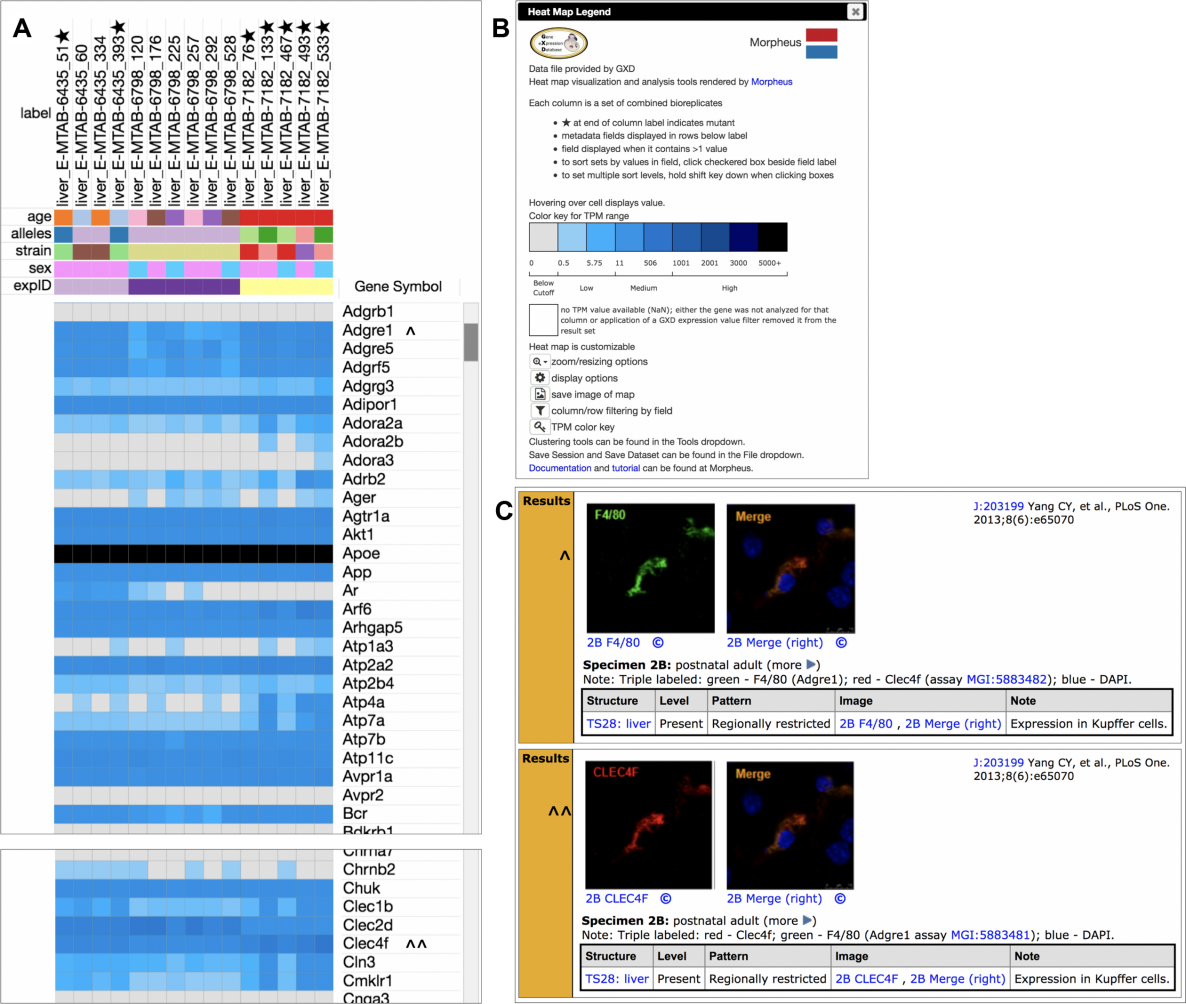
Heat Map of RNA-Seq Results. Expression heat map of RNA-Seq data from GXD expression results shown in Figure 2, rendered using the Morpheus tool. (**A**) Heat map grid shows quantitative expression of genes (rows) from distinct bioreplicate sample sets (columns). Column labels are derived from anatomical structure, experiment ID and bioreplicate set ID. Stars indicate mutant samples, wild type samples have no star. Common annotations for a given metadata type (age, strain, sex, etc.) share the same color in that row (if all samples share the same metadata value, that row is not shown, by default). Expression data rows (Gene Symbol) reflect average, quantile-normalized TPM values for the corresponding genes in each bioreplicate set, using a color scheme designed to accommodate the wide dynamic range of TPM values in the data (see B). Cell values (metadata or TPM) are displayed by hovering over the cell. Genes *Adgre1* and *Clec4f* are marked (carets) for comparison in (C). (**B**) Heat map legend includes TPM range color key, guide to usability options (sorting, filtering), pointers to data clustering tools and file saving, and links to documentation at the Morpheus resource. (**C**) Partial GXD assay results (immunohistochemical staining) for two genes included in the RNA-Seq heat map (carets in A) reveal coexpression of these genes in Kupffer cells.

Figure 2C features assay results (partial) for the filtered search in Figure 2B, showing integrated expression data from RNA-Seq and Immunohistochemistry assays. Results for two of the 504 genes are shown (*Adgrb1* and *Adgre1*). New metadata columns were added to the Assay Results tab to distinguish bioreplicate sets (toggled open in Figure 2C). RNA-Seq data-specific columns include TPM Level, the number of biological replicate samples and a Notes field used to record salient metadata features not accounted for under other metadata fields. Each RNA-Seq data row in the table represents the expression record (with averaged QN TPM value mapped to a TPM Level) from that set of combined bioreplicate samples for the corresponding experiment. RNA-Seq rows also feature novel links to the Expression Atlas, which offers additional display and analysis tools, and to the GXD RNA-Seq and Microarray Experiment Summary for the corresponding experiment. All RNA-Seq results in the table can be viewed in a heat map rendered by the Morpheus resource (https://software.broadinstitute.org/morpheus/) (discussed below), and all expression results from the user's search can be exported to a text file for further analysis elsewhere. To illustrate the benefits of integrated expression results from RNA-Seq and classical expression assays, a link to GXD assay details in the Images column for one of the immunohistochemistry assays is indicated (detailed in Figure 4C). The other summary views (Genes, Assays, Images, and the Tissue × Stage and Tissue × Gene Matrix tabs) are described elsewhere (8). Slight modifications were made to the Assays tab and the Tissue x Gene Matrix to accommodate the whole genome perspective of RNA-Seq data. For the Assays tab, instead of listing all genes for an assay (as done for classical assay types), each RNA-Seq experiment returned is displayed in a single row, with the option to filter the expression result set by that experiment (Figure 3). Links are also provided to the RNA-Seq and Microarray Experiment summary page for each RNA-Seq experiment. For the Tissue × Gene Matrix, column pagination (genes) was introduced to manage display of the vast number of genes that can be included in RNA-Seq results (not shown).

### Morpheus heat map of GXD RNA-Seq data

We have embedded the Morpheus heat map visualization and analysis tool from the Broad Institute (https://software.broadinstitute.org/morpheus/) into the GXD interface. When RNA-Seq results are present, the expression results summary page provides a link to a Morpheus heatmap (see Figure 2C). This gives users one-click access to the wide range of display and analysis tools offered by the Morpheus resource for RNA-Seq data sets tailored by GXD’s powerful search and filtering functions. The Morpheus heat map rendered for the RNA-Seq results from the GXD query in Figure 2 is shown in Figure 4. The heat map displays quantitative expression (using a color-coded average QN TPM value range) for each gene (rows) and the corresponding biological replicate sample sets (columns) returned from the GXD query (Figure 4A).

Columns in the heat map grid represent biological replicate sets. Column labels are derived from the anatomical structure, experiment ID and GXD-assigned bioreplicate set ID of featured biological replicate sets. Stars shown in column labels indicate that the samples were derived from mutant mice, which is important when interpreting expression profiles. Wild type samples have no star in the column label. Morpheus provides column data display tools to help users recognize patterns in the data, including colored metadata rows just below the column labels. The tool assigns separate colors to distinct metadata annotations in each metadata row making it easy to spot common annotations across the sets of bioreplicates (underlying values are displayed on mouse-over). By default, Morpheus suppresses display of metadata rows that are shared by all columns, but users can include these common rows if desired. Morpheus offers primary and secondary metadata sorting by simply clicking on metadata row labels, while additional sorting and filtering options are available (for columns and rows) via the main display tools (see Figure 4B).

Rows in the heat map grid represent genes. The default label for gene rows is Gene Symbol, MGI ID and Ensembl gene ID (reduced to Gene Symbol in Figure 4A). Default TPM value color code ranges (shown in the heat map legend in Figure 4B) were designed to approximate display settings used by the Expression Atlas, facilitating comparison between resources, and to manage the enormous dynamic range of RNA-Seq data (color saturation set at 5000 TPM). Morpheus display options allow users to adjust the colors and TPM ranges of the heat map, to improve resolution in any TPM value range desired. The TPM value for any cell is displayed by mouse-over. It is possible to generate a heat map that has some empty cells; these are colored white and display ‘NaN’ (Not a Number) on mouse-over. This can happen when an expression value filter (TPM Level or Detected?) was applied to the GXD result set before rendering the heat map (since genes can have a range of TPM values across the bioreplicate sets present). This can also happen when an experiment has fewer total gene annotations (if not updated to the latest Ensembl build, for example). The ability to align metadata values with TPM value ranges allows users to spot biologically significant expression profiles, such as sex- or strain-specific effects and expression effects of specific mutants compared to wild type samples. Morpheus also offers a range of data clustering tools under the Tools dropdown menu (not shown). To keep response times manageable, we’ve imposed a 10 million result limit for export to the heat map. A processing progress bar is displayed while the heat map is generated.

An example of the value of integrated RNA-Seq and classical expression assays is shown for genes *Adgre1* and *Clec4f* in Figure 4A and C (marked with carets for comparison). GXD assay results of immunohistochemical staining in mouse liver reveal spatially-restricted coexpression of these two genes in Kupffer cells (which are resident liver macrophages), while the RNA-Seq heat map provides a quantitative expression survey across the bioreplicate samples included.

## FUTURE DIRECTIONS

ArrayExpress originally imported all data sets present in GEO. In fact, 2621 of the 3156 experiments in GXD’s metadata index were originally submitted to GEO. Because ArrayExpress has stopped importing GEO data, we will implement a direct data load from GEO to supplement our load from ArrayExpress. Our goal is to provide a complete, non-redundant index of GXD-relevant RNA-Seq (bulk and single cell) and microarray expression experiments from both repositories, with associated biological source metadata properly annotated and searchable.

We will continue to integrate RNA-Seq data from the EBI Expression Atlas that provide GXD a quantitative perspective of endogenous gene expression from wild-type and mutant mice, covering the complete life span of the laboratory mouse. While our literature curation efforts for classical expression data types have focused on studies of gene expression during embryonic development, and will continue to do so, we have always accepted direct submissions of postnatal expression data. With the addition of RNA-Seq data, our coverage of postnatal expression information has now increased significantly.

The advantages of integrating RNA-Seq expression data into GXD extend beyond the detailed quantitative perspective they provide (compared to RNA *in situ* or immunohistochemistry experiments). As a whole-genome method, RNA-Seq brings more comprehensive information about the absence of gene expression in specific tissues compared to *in situ* studies. For *in situ* studies, a single section can demonstrate gene expression, but extensive serial sectioning is required to show lack of expression, and this information is often not reported. GXD’s Differential Expression Search takes this sparsity of information about absence of expression into account. The algorithm for searches such as ‘What genes are expressed in liver and not anywhere else?’ relies mostly on positive expression results, i.e. the search will find genes for which there is evidence of expression in liver and no evidence of expression in any other structure [see (2) for more detail]. We will revise this algorithm to take advantage of the extensive absence of expression information provided by RNA-Seq experiments. Including RNA-Seq data in differential expression searches will expand the utility of these searches, and we plan to leverage this utility by providing additional support for differential expression analyses in general.

We plan to expand the accessibility and benefit of cell type information in GXD by including annotations to the Cell Ontology (19) in our expression records. Such information is currently recorded in notes fields (as indicated in Figure 4C). The combined use of terms from the anatomy ontology and the cell ontology will refine our standardized representation of expression patterns and add new search capabilities. This representation also enables future integration of single-cell RNA-Seq (scRNA-Seq) data, for which GXD can provide important tools for further interpretation and analysis. For example, the marker sets that distinguish cell clusters discovered through scRNA-Seq studies must be analyzed further, such as by *in situ* experiments, to confirm and characterize potential new cell types. GXD currently records RNA *in situ* and immunohistochemistry data, including data published in conjunction with scRNA-Seq experiments, thus we are well positioned to support gene/marker-driven discovery of new cell types. This illustrates the value of analytical cross-inspection of different expression data types, facilitated by GXD’s integrated data platform.

## USER SUPPORT

GXD provides support to its users through dedicated User Support personnel, detailed on-line documentation and quick tutorials. User Support can be contacted via email at mgi-help@jax.org or by clicking the ‘Contact Us’ link in the navigation bar at the top of all web pages. Upon request User Support will provide remote interactive training sessions and on-site visits. The online documentation can be accessed by clicking on the question mark in the upper corner of most pages. Quick tutorials (and links to other informational material) can be found on the Help tab of the GXD home page (http://www.informatics.jax.org/expression.shtml).

## CITING GXD

The following citation format is suggested when referring to data downloaded from GXD: These data were retrieved from the Gene Expression Database (GXD), Mouse Genome Informatics, The Jackson Laboratory, Bar Harbor, Maine, USA (URL: http://www.informatics.jax.org) on [date (month, year) when you retrieved the data cited]. To reference the database itself, please cite this article.
